# Mild Encephalitis/Encephalopathy With a Reversible Splenial Lesion (MERS) in an Adult: An Associated Finding in Severe Acute Pancreatitis With Multisystem Involvement

**DOI:** 10.7759/cureus.84089

**Published:** 2025-05-14

**Authors:** Nada Nfaoui, Mina Aallam, Safae Zahlane, Mohamed Chraa, Nissrine Louhab

**Affiliations:** 1 Neurology, Mohammed VI University Hospital, Marrakech, MAR; 2 Medicine and Pharmacy, Cadi Ayyad University, Marrakech, MAR

**Keywords:** acute inflammatory response, adult mers, case report, multisystem involvement, reversible splenial lesion syndrome

## Abstract

Mild encephalitis/encephalopathy with a reversible splenial lesion (MERS) is a rare clinico-radiological syndrome characterized by transient lesions of the splenium of the corpus callosum, typically occurring in the context of infections. While most frequently observed in children, adult cases remain infrequent and underreported. We present the case of a 30-year-old male patient with a preceding viral-like illness who was admitted for acute pancreatitis, renal failure, and neurological symptoms including confusion, dysarthria, ataxia, and postural tremors. An MRI of the brain revealed a reversible lesion in the splenium of the corpus callosum. Admission blood workup showed systemic inflammation, elevated inflammatory markers, and cytopenias, except cerebrospinal fluid (CSF) analysis and infectious serologies were unremarkable. The patient required hemodialysis and supportive management, including antibiotics, with clinical and radiological recovery of both the MERS and the other systems involved. This case highlights an atypical adult presentation of MERS associated with severe multisystem involvement, highlighting the importance of recognizing this syndrome in adults with systemic infectious syndromes and neurologic symptoms. Awareness of such presentations is essential for accurate diagnosis, timely intervention, and favorable outcomes.

## Introduction

Mild encephalitis/encephalopathy with a reversible splenial lesion (MERS) is a clinical and radiological spectrum disorder called “reversible splenial lesion syndrome.” The splenium is the posterior part of the corpus callosum. Functionally, the corpus callosum allows the transfer of information from one hemisphere to the other and coordinates the responses of each hemisphere. Its anterior part, connecting the prefrontal cortices, is involved in cognitive functions, while its posterior, splenial part is involved in sensory functions. The usual clinical neurological features of MERS are mild central nervous system disorders such as seizures, confusion, and delirium. Other neurological symptoms include motor deterioration, slurred speech, neck stiffness, coma, tremor, ataxia, somnolence, dysarthria, visual disturbance, and dizziness. Affected patients usually recover completely without any sequelae within a month after the onset of neurological symptoms [1]. T2-weighted and fluid attenuated inversion recovery (FLAIR) brain MRI sequences show hyperintensities without contrast enhancement in the center of the corpus callosum, which resolve completely [2]. Usually, MERS is seen in children and is much less frequent among adults [3]. We describe a case of MERS in an adult with pancreatitis and severe kidney failure and review the literature on MERS in adults.

## Case presentation

This is the case of a 30-year-old male patient admitted to the ER with the sudden onset of epigastric pain, melena, and kidney failure, preceded by flu-like symptoms a week before. On admission, the patient was drowsy and in a state of confusion. His past medical history was unremarkable, and there was no history of past or recent drug use or alcohol intoxication. Neurological examination revealed dysarthria, limb ataxia, and postural tremors. In addition, he presented generalized purpura.

Blood workup upon admission showed normochromic normocytic anemia, an elevated leukocyte count with bi-cytopenia (lymphocytes and platelets), and elevated serum markers of inflammation. There was further alteration of kidney function (creatinine 121.2 mg/l, blood urea nitrates 3.87 g/l). Infectious serology for human immunodeficiency virus (HIV), hepatitis B and C viruses, and syphilis was negative. Cerebrospinal fluid (CSF) analysis was normal (Table 1).

**Table 1:** The patient's blood workup on admission

Values	Observed values	Normal range
Hemoglobin	8.5 g/dL	13-17 g/dl
Platelets	108000 uL	150 000-450 000 uL
Lymphocytes	770 uL	1000 -4000 uL
White cell count	20800 uL	4000-10000 uL
Ferritin	3903 ng/ml	30-400 ng/ml
Erythrocyte sedimentation rate (ESR)	87 mm/h	00-10 mm/h
Creatine kinase (CK)	13502 UI/L	39-308 UI/L
Neutrophils	16510 Ul	2000-7500 Ul
Urea	3.87 g/l	0.25-0.48 g/l
Creatinine	121.2 mg/l	07-12.00 mg/l
Human immunodeficiency virus (HIV)	Non-reactive	Non-reactive
Hepatitis C virus (HCV)	Non-reactive	Non-reactive
Hepatitis B surface antigen (HBsAg)	Non-reactive	Non-reactive
Venereal disease research laboratory (VDRL)	Non-reactive	Non-reactive
Aspartate aminotransferase (AST)	96 U/L	10.00-50.00 U/L
Alanine aminotransferase (ALT)	74 U/L	10.00-41.00 U/L
Alkaline phosphatase	95 U/L	40-129 U/L
Gamma glutamyl transferase (GGT)	70 U/L	10.00-71.00 U/L
C-reactive protein	155 mg/dL	<6 mg/dL
Lactate dehydrogenase	691 U/L	0 - 250
Lipase	567 U/L	13 - 60
Prothrombin rate	54.2%	70%-100 %
International normalized ratio (INR)	1.42	1.00
Activated partial thromboplastin time (aPTT)	23.5 sec	30 - 35 sec
Mean corpuscular volume (MCV)	81.9 fL	80-100 fL
Mean corpuscular hemoglobin concentration (MCHC)	34 g/dL	32-35 g/dL

**Figure 1:**
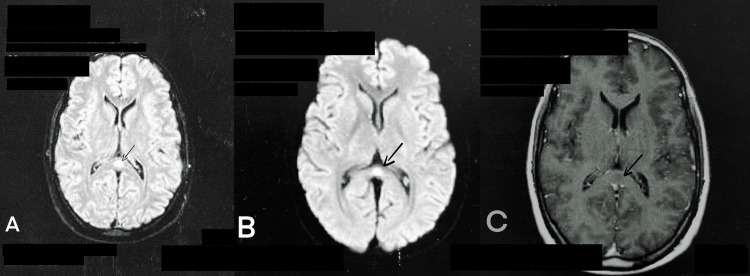
Brain MRI obtained on admission (axial view) (A) A hyperintense lesion in the splenium of the corpus callosum (SCC) on fluid‐attenuated inversion recovery images (FLAIR) (black arrow); (B) A hyperintense lesion in the SCC on diffusion-weighted imaging (DWI) (arrow); (C): no contrast enhancement on T-1 weighted images (arrow)

Brain MRI revealed one nodular lesion in the splenium of the corpus callosum (SCC). It was hypointense on T1, hyperintense on T2-weighted images, fluid-attenuated inversion recovery images (FLAIR), and diffusion-weighted images (DWI) and without contrast enhancement (Figure 1).

Workup for gastrointestinal symptoms revealed stage A pancreatitis on abdominal CT scan. There was also extensive wall thickening of the colon, suggesting an inflammatory origin. Thoracic CT revealed pneumomediastinum and micronodules likely of infectious origin, and no notable findings on the abdominal ultrasound.

**Figure 2:**
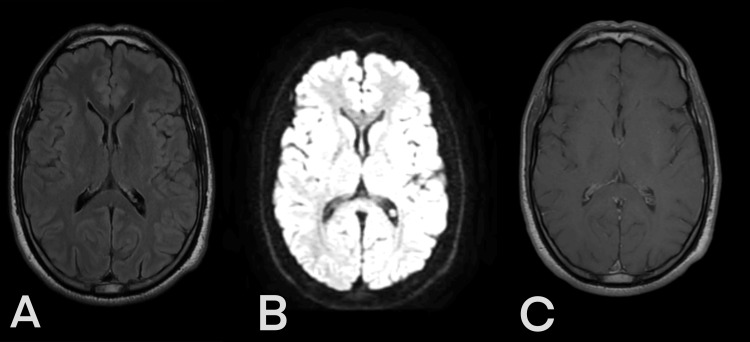
Brain MRI (axial view) obtained two months after the onset of the condition (A) Fluid‐attenuated inversion recovery image (FLAIR); (B) Diffusion‐weighted image (DWI); (C) T1‐weighted imaging

After conditioning and blood transfusion, the patient underwent hemodialysis and was placed on dual antibiotic therapy for the pancreatitis. The overall evolution was favorable, with the improvement of both neurological and extraneurological symptoms. Cerebellar symptoms resolved within one month. Brain MRI performed two months after the onset of the condition revealed complete disappearance of the splenial lesion (Figure 2), and a second thoracic and abdominal CT done after two months also came back normal. The diagnosis of MERS was retained.

## Discussion

It is a clinical-radiological neurological condition typically found in extraneurological infections. MERS is rare in adults. The novelty in the present case is the association with an infectious syndrome with systemic inflammation and multiorgan involvement.

The main neurological manifestation in our case was drowsiness and cerebellar involvement (dysarthria, ataxia, and postural tremors). According to a systematic review of 51 patients, neurological manifestations were preceded by prodromal symptoms in most patients (88%), with fever (78%) being the most common. Headache was the most common neurological symptom (50%), followed by seizures (22%) and altered consciousness (22%). Inflammatory changes in CSF were present in half of the patients [4]. Other general clinical symptoms include digestive tract disturbances (vomiting and diarrhea) [5, 6].

In this case report, the cause was probably a viral infection based on the patient's history and the fact that no germ was identified in our investigation. In 87% of the literature, MERS was found to occur in the context of an infection. Various germs (mainly viruses but also bacteria or parasites) can trigger MERS, but they failed to be identified in around 50% of the cases in a series by Grosset et al. [7]. It remains unclear why reversible splenial lesions selectively occur in the SCC, despite numerous pathology and neuroimaging studies. It could be that the specific affinity of viral antigens or induced antibodies to the splenial axonal receptors is responsible for the splenial involvement in viral encephalitis. Moreover, it has been speculated that the SCC has a specific vulnerability to excitotoxic injury in metabolic diseases, which makes this area selectively involved in different pathological events [8-11].

Typical MRI features are reversible hyperintense signals on T2-weighted images, FLAIR, and DWI. In addition, the apparent diffusion coefficient (ADC) of the lesion is decreased on ADC maps, and hypo- or isointense signals may appear on T1-weighted imaging sequences without contrast enhancement [1, 12]. The reversibility of lesions defines the syndrome and confirms the final diagnosis. This was the case with our patient.

Neurological symptoms are expected to disappear within a month, and isolated reversible lesions in the SCC are a good prognostic marker for a benign disease course in affected patients [3]. Although it is described as mild and reversible, the outcome isn’t always benign, as impaired consciousness or other system involvement may shift the prognosis to a more dire one, including death as a probable outcome. In contrast, there have been case reports of patients who presented with only fever or headache and without neurological symptoms [1].

Further research is required to better understand the mechanisms of MERS so that we can do better at predicting the disease's evolution and prognosis, all of which will help us give efficient care to the patient.

## Conclusions

This case is a reminder of the possibility of MERS in adults and its possible association with severe multisystem disease. It is a clinical-radiological disorder that can manifest on a spectrum from headache to altered consciousness. The severity in our patient, other than his confusional state, entailed the extra-neurological involvement. This points to the importance of the associations or context of diagnosis of MERS as determinants of outcomes. This context should be clearly identified to allow for prompt, specific treatment of associated conditions. This may require exhaustive workup and collaboration with other specialties to manage patients, as was the case with our patient. While this case is a reminder of the possibility of MERS in adults, it should also serve as a reminder of the need for more work on the subject to better characterize the condition and help understand the underlying mechanisms of its occurrence.
